# Belladonna an Antidote to Opium

**Published:** 1869-02

**Authors:** 


### Correspondence.

### Belladonna an Antidote to Opium.

In the *Buffalo Medical and Surgical Journal*, there is a case reported in which belladonna or atropia was successfully used as an antidote to opium or morphine poisoning. That belladonna is, to a great extent, an antidote to such poisoning, I know from no small and untrustworthy experience, but from repeated and reliable facts. Having been several times called in consultation, in cases of opium poisoning, and, in such cases, found in each instance the attending physician was entirely ignorant of the fact that belladonna possessed any such antidotal power.

As many as fifteen years ago, from observing the opposite effects upon the pupil of the eye, from the above remedies—belladonna enlarging and opium contracting the pupil—I conceived the idea that the condition of the pupil of the eye should be the diagnostic indication for the use of these remedies respectively, in doubtful cases. Proceeding upon this idea, I was well satisfied with the result, and have ever since made the pupil of the eye my guide in practice in regard to these remedies. Soon after I conceived the idea that these remedies might be used, the one an antidote for the poisoning of the other. Experiments, which it is unnecessary here to detail, confirmed the correctness of this idea, and I had not long to wait for opportunities to test it in practice. I supposed, at the time, the idea was entirely original with me, as certainly it was, but I am not quite sure but others had used the remedies with similar intent prior to that time.

I published my views and experience at different times and in different journals, during the succeeding five or six years. I am yet confident that the fact of the antidotal and dissimilar action of these two remedies is not as well and widely known as it deserves to be. Neither do I believe that the condition of the pupil of the eye of a patient, in any disease, is sufficiently made an indication for the use of these remedies. He who gives opium in any disease, when the pupil is greatly and abnormally contracted, will, if he is at all observing, have occasion to regret it.

Dr. E. Williams, of Cincinnati, Ohio, has experimented quite largely with these remedies, and, in the January number of the *Lancet and Observer*, for the current year, he has the following practical remark: "Morphia, although an antidote to atropia in ordinary cases where water is freely allowed, is hardly an antidote where fluid is entirely withheld."

In the *Medical Times and Gazette*, Prof. A. Von Græfe has the following on the antagonistic action of opium and belladonna: "When a solution of atropine has been injected hypodermically, three or four minutes afterwards the pupil becomes dilated, the pulse rises to 140–160, and other symptoms of narcosis by atropine are observed. If morphia is then injected, all these phenomena, which would otherwise last for hours, disappear in a very short time. After a hypodermatic injection of morphia, a considerable myosis is observed, and the pupil cannot be dilated."

Again he says: "Opium and belladonna have an antagonistic effect upon the muscular fibres of the tensor chorividiæ, as upon the muscles of the iris; and the analogy would be quite complete, if a double and antagonistic innervation of the tensor chorividiæ, by both the third pair and the sympathetic nerve, was just as certain as it is for the iris."

It is too much to expect that belladonna or atropia, will invariably prove a successful antidote to opium or morphia, in all cases and in all hands. There is, in the use of these remedies, a very great opportunity for the exercise of judgment and discretion, the one as an antidote to the other. The dose of the one should be proportionate to the dose of the other—the antidote to the poison. If a poisonous dose of opium has been taken by the mouth, and has been so long taken as to have wholly entered the system, a dose of belladonna by the mouth may fail, or seem to be ineffectual, because the patient may die before the antidote is made available by its proper entrance into the circulatory system. It is a well known fact that medicines, hypodermatically applied, act much sooner than when taken into the stomach. Hence, when the poisonous operation of the one is in full operation, and the speedy action of an antidote is desired, the other should be brought to bear by hypodermatic injection.

O. C. Gibbs, M. D.,
Frewsburg, Chautauqua Co., N. Y.

P. S. The above was written before the January number of the *Buffalo Medical and Surgical Journal* came to hand, in which extracts are made, from a paper by my friend, Dr. Samuel R. Percy. Neither have I seen Dr. Percy's paper. From extracts in the *Journal* I see Dr. Percy quotes also, and more fully, from Dr. A. Von Græfe.

Dr. Percy was at my house, seven or eight years ago, and I remember well our conversation upon this subject. For the last six or seven years my health and other business connections have precluded the possibility of my keeping myself posted thoroughly in medical journal literature. On renewing my professional journal reading, it seems a little surprising to learn that this subject is just beginning to agitate the medical world.

I have no ambition for the questionable honors of originality of suggestion, even had I the right to claim them, which I doubt, and my only object in writing now is, to add the small weight of my evidence in favor of an important therapeutical fact, which I have long since considered well established. O. C. G.

